# Bugging Forecast: Unknown, Disliked, Occasionally Intimate. Bed Bugs in Germany Meet Unprepared People

**DOI:** 10.1371/journal.pone.0051083

**Published:** 2013-01-02

**Authors:** Conrad Seidel, Klaus Reinhardt

**Affiliations:** 1 Animal Evolutionary Ecology, University of Tübingen, Tübingen, Germany; 2 Department of Animal and Plant Sciences, University of Sheffield, Sheffield, United Kingdom; Queensland Institute of Medical Research, Australia

## Abstract

Bed bugs appear to be feared more than vector insects and other household pests. The reasons for this exaggerated fear are not fully understood. One hypothesis is that the folk knowledge on recognising and controlling bed bugs decreased as bed bugs became rarer in the 1960s and led to irrational perceptions. Here, we examine people’s ability to recognise a bed bug and their response what to do in case of an infestation. We found that 13% of a sample of 391 people in four large German cities recognised a bed bug; 15% of all respondents would call a pest controller in case of bed bug infestation. This results in the pessimistic estimate that 97% of all early-stage infestations could go untreated. We discuss additional scenarios. The effectiveness of efforts to educate people about the presence of bed bugs has never been tested, but our sample is useful to guide future studies. We found three sources of information were associated with increased recognition rates of bed bugs: a) previous contacts with bed bugs (60% recognition), b) knowledge from friends or relatives (25%) and school or education courses (15%). By contrast, people who heard of bed bugs from television, print media or the Internet showed reduced recognition rates. We propose that the former factors be tested for educational interventions. In Germany, the bed bug is an estranged creature to many people, a fact that seems to hinder rational approaches to their control.

## Introduction

The relationship between humans and bed bugs (particularly the common bed bug, *Cimex lectularius* L.) has lasted for at least four millennia. The earliest *Cimex* records that most likely are *C. lectularius* are from villages around the Egyptian pyramids [1] and from Roman excavations sites [2]. Bed bugs have also persisted from ancient times in the written literature as in Aristophanes’ *Clouds,* in the oral literature [3] as well as in folklore [4]. Various aspects of bed bug control have recently been reviewed [4]–[6]. Along most of their joint path with humans, bed bugs have been viewed as an unavoidable nuisance; a pest that has to be lived with. Currently, the number of articles on bed bug occurrences is rising (reviewed in [6]). However, the lack of previous monitoring means that most of these studies provide anecdotal, rather than scientific evidence that the number of bed bug infestations is rising (reviewed in [7]–[9], but see [10], [11]). Bed bugs seemed to be increasingly viewed as intolerable and are classified as urban pests and a potential health hazard [12]. The presence of bed bugs can harm a hotel’s reputations leading to possibly serious economic consequences. However, quantitative data on the damage are largely lacking. Such damage does not seem to occur with the presence of other blood-sucking insects, or cockroaches. Bed bugs do not transmit diseases [6], [13] but can cause skin irritations [14], and in rare, largely over-reported cases, stronger dermatological responses [15] as well as psychological stress [16]. With the possible exception of psychological stress, the evidence basis for the clinical relevance of bed bugs is limited [6], [13]. However, the reasons for the psychological effects and why people seem to fear bed bugs much more than even disease vector insects such as mosquitoes or fleas, is largely unexplored.

Most reports agree that throughout the 1960s and 1970s the rate of bed bug infestations was very low, following a constant decline from the 1930s onwards [9]. This relative rarity of infestations may have lead to ever fewer people having contact with bed bugs and recognizing them but empirical data are rare. For example, only 10% of 358 persons in England recognized a bed bug when shown one in a test tube [17]. In the US, 40% of the people who had an active infestation did not realise it [18]. It is possible that the ‘detachment’ of bed bugs from the daily life of humans has generated uncertainty of how to avoid a bed bug infestation and how to avoid in infestation becoming entrenched (see [19], [20] for examples). This uncertainty may cause or reinforce the fear of bed bugs and be a major contributor to the psychological stress reported by Goddard & deShazo [16]. By contrast, familiarity with an animal may reduce irrational feelings. For example, Majekodunmi et al. [21] found more rational views among tenants living in cockroach-infested houses than among tenants without a cockroach infestation. Randler et al. [22] report that school children exhibited less fear and disgust of woodlice after physical contact with them.

In some countries substantial efforts are undertaken to educate people about the presence of bed bugs, or to sell products to control them, often on a multi-media basis. However, the effectiveness of such efforts has never been tested, which might be critical because a huge, detailed picture or advertisement showing a bed bug may not lead to people recognizing the 4–5 mm large, lentil-sized adult insects in real life. An alternative measure could be to specifically educate staff of the hospitality and travel industry by providing general information about bed bugs and by showing them live bed bugs and their traces. This approach rests on the untested assumption that people can remember bed bugs once they had them or had seen them.

We interviewed people for a research project aiming to compare different methods of surveying bed bug abundance in Germany [23]. Based on these interviews we here explore people’s knowledge about bed bugs. We aim to answer the question how educational interventions could be effective to improve knowledge. We will conclude that the bed bug currently is an estranged creature to many people, a fact that seems to hinder rational approaches to their control.

## Methods

Interviews were carried out in four large (>500,000 inhabitants) German cities (Cologne, Hamburg, Leipzig, Munich). This choice reflected German geography (west, north, east, south, respectively) and the existence of long-term data sets on bed bug abundance for two of the cities (to be analysed separately). All interviews took place in the city centre, specifically in pedestrian zones, shopping arcades, public squares, and a waterside promenade. Consent was not requested because our study was an epidemiological study with anonymous, non-personal data for which ethical approval is not required according to the law of the regional government of Baden-Württemberg.

**Figure 1:**
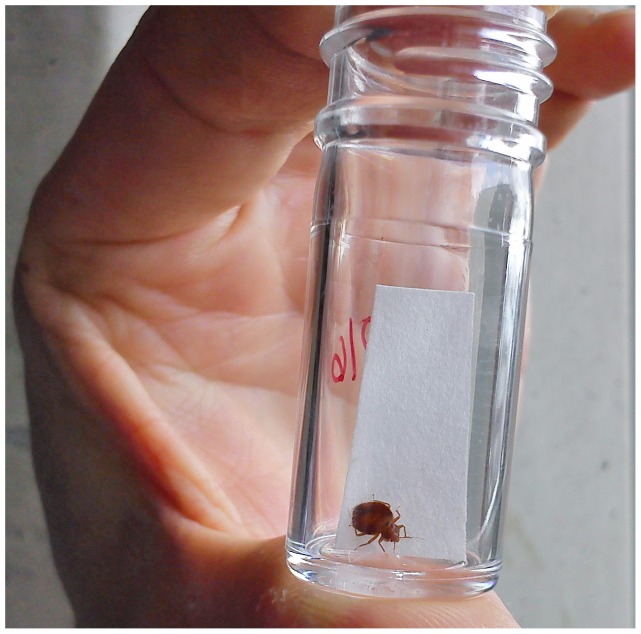
Male bed bug in a test tube as presented to people in an inquiry about bed bugs.

One of us (C.S.) presented a male adult bed bug *C. lectularius* (Figure 1) to nearly 100 people in each city (total 391). People were asked whether they knew what animal it was, and what they thought it was. After revealing the identity of the insect, the people were asked whether they knew what to do in case of an infestation. Finally, they were asked “Did you ever have contact with bed bugs?” and age and gender of the respondents were noted. People aged between 6 and 92 and a similar in each of five 15-year age cohorts were chosen [17]. Forty-three per cent of the respondents were male. A detailed analysis of the age-related pattern is in progress but outside the scope of the present paper. In this paper we ask what people think they were shown and whether this would potentially support or hinder the control of an infestation.

The responses of people who had had contact with bed bugs suggested their contact arose from an infestation. Therefore, the proportion of people correctly identifying bed bugs among people who had contact with bed bugs indicates how effective it would be to present people with a bed bug as an educational intervention.

## Results

### Recognising Bed Bugs

**Figure 2:**
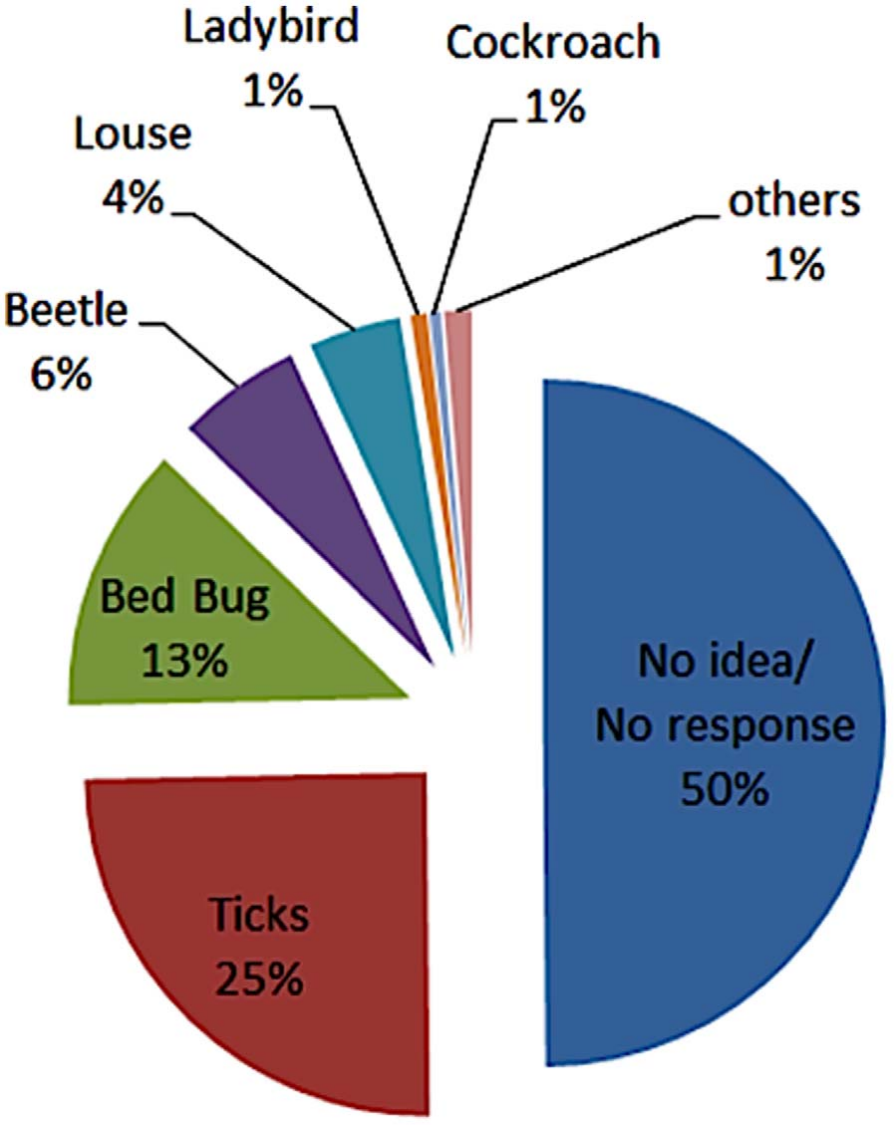
Responses by 391 people that were shown a live bed bug and asked about the identity of the animal. The bed bug was presented in a test tube (see Figure 1). People aged 6–92 years.

87.5% of the people were unable to identify the bed bug (average age 38 years), 12.5% were able to (average age 48 years) (Figure 2). There was no difference between male and female respondents who correctly identified the bed bug (12.6% and 12.5%, respectively).

**Table 1:** Sources of information people in Germany know bed bugs from, the proportion of each category that correctly identified the bed bugs and the age-corrected prediction of that category.

Category	Citing as source of information	Correctly identified bed bug	Average age namingit as only source	Predicted correct identificationbased on that age
“general”	25.3%	11%	43	13%
friends, relatives, childhood	20.8%	25%	52	15%
TV	31.1%	10%	33	10%
own contact	6.9%	59%	53	15%
school/education	4.8%	15%	25	8%
print media and internet	13%	8%	38	12%
others	4.1%	11%	42	13%
**Whole sample**		**13%**	**41**	

Fifty per cent of the interviewees had no idea what they were presented with or, occasionally, did not want to reveal their thoughts. One quarter suggested it to be a tick (Figure 2). Three people stated they recognized a ladybird (all of them adults - aged 18, 43 and 50). The breakdown of responses (Figure 2) also indicates that many people cannot correctly recognize ticks, cockroaches or lice. The term “beetle” was considered incorrect despite the superficial resemblance with bed bugs. Other responses included mite (2), woodlouse (2), scale insect (1), and cockchafer (1).

### Knowing about Bed Bugs

85% of people had heard of bed bugs, with almost 100% of over 60-year-olds. Of the people who had heard about bed bugs, most did so from television, friends or relatives, or classified their source as unspecified “general knowledge” (Table 1). Cultural knowledge transmission (i.e. ‘word of mouth’) by friends or relatives was important; of the 61 people who said relatives or friends to be their only source of information, 15 (25%) recognized a bed bug, which is significantly higher than the nine out of 86 TV-only informers (10%) (Fisher’s exact test, p = 0.026), but not from the 2 out of 13 school/university-only source (15%) (Fisher’s exact test, p = 0.719). Surprisingly, only 2% specifically named the internet as the source of knowledge.

Of the people who had previous contact with bed bugs (n = 27), a majority (59%), but not all, correctly identified bed bugs (Table 1). Most other of these respondents referred to ticks. Thus, of people who had not have bed bugs (364), only 8.8% identified them correctly, an almost 7 fold difference (Fisher’s exact test, p<0.0001).

Some of these comparisons may be hampered by the age structure in the respective respondent groups. Predicting the recognition ability using a linear regression over age (C. Seidel and K. Reinhardt, unpubl. data) suggests that i) personal experience with a bed bug infestation, ii) reports by friends and relatives and, to a lesser degree, iii) school/education leads to higher recognition than would be expected from a random group of the same age (Table 1). These three measures may be viewed as candidates for effective interventions. Other sources of information seem non-effective means of intervention (Table 1).

Learning about bed bugs as a result of experiencing an infestation, appears to be a long-lasting effect: the last time the respondents had contact with bed bugs did not differ much between those that did recognize bed bugs (35 years ago) compared with those that did not recognize them (30 years ago). One person recognized a bed bug although he stated to have seen his last one in the 1920s (excluding this person, gives an average of 32 years ago for people who recognized bed bugs.

Seventeen of the 27 respondents (63%) had their contacts with bed bugs in Germany, six outside central Europe.

### Getting Rid of Bed Bugs

**Figure 3:**
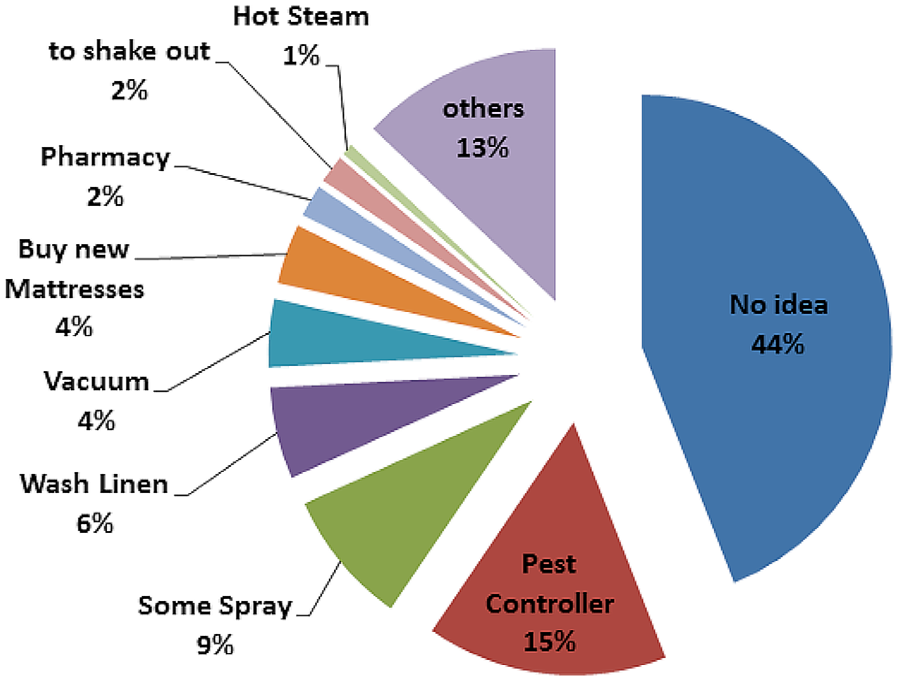
Responses by 391 people that were shown a live bed bug and asked what they think should be done in case of a bed bug infestation. Respondents were the same as for Figure 2.

Upon the question “How do you think you can get rid of bed bugs?”, most interviewees had no answer at all. Only 15% opted for possibly the only effective solution to call a professional pest controller (Figure 3). Assuming people would get correct advice in the pharmacy, an extra 2% may be added. A mere eight people (2%) stated they would inform themselves on the Internet. These might also come to the conclusion to hire the services of a professional pest controller (19%).

No data exist of how effective other, frequently-promoted control measures are to eradicate an existing, small infestation, beyond using a pest controller. However, the categories “to shake out the beddings”, “buy new mattresses” “place bed outside” and “wash the mattress” “wash myself” and some of the “others”? (together comprising 14%) are entirely ineffective measures. One sixth of respondents suggested “vacuuming”, “pharmacy”, “some spray” or “hot steam” (16%), which are proposed measures that can help to reduce the biomass of a bed bug infestation, or control small infestations. However, all these measures can potentially also make the situation worse by causing bed bugs to disperse into other rooms or apartments, even as a result of vacuumed bugs being disposed of improperly.

In summary, given correct advice in a pharmacy, if dispersal of the infestation was to occur as a result of washing or changing the mattress, vacuum cleaning, changing the bedding, or spraying something on the bed bugs, then 54 of 172 (31.4%) would potentially apply measures that could make an infestation worse.

Twenty-two per cent of the people recognizing a bed bug suggested to call a pest controller. This is a non-significant difference to the 13% of the people who would also do so but had not recognised it (Fisher’s exact test, p = 0.124; excluding people who did not provide an opinion at all, 31 vs. 23%: p = 0.289). The proportions were also non-significantly different for people in both groups that suggested measures that had the potential to make the infestation worse (29 and 34%, Fisher’s exact test, p = 0.633; excluding people who did not provide an opinion at all, 43% vs. 61%: p = 0.060).

People who had previous contact with bed bugs would not call the pest controller more frequently (19%) than people without contact (13%) (Fisher’s exact test, p = 0.378; excluding people who did not provide an opinion at all, 26 vs. 24%: p = 0.788). Likewise, people with prior contact recommended measures that could make infestations potentially worse at a similar rate (46%) than people without prior contact (35%) (Fisher’s exact test, p = 0.292; excluding people who did not provide an opinion at all, 63 vs. 65%: p = 1.00).

## Discussion

We found that most people knew that bed bugs exist but few recognized one. Prior exposure, information by friends and relatives and education seemed to increase the recognition ability. Few immediately knew what to do about an infestation. Prior exposure did not lead people to suggest measures that would increase the chance of eradicating the infestation. Below, we discuss the three levels of human intimacy with bed bugs.

### Recognising Bed Bugs

The total proportion of people recognizing a bed bug in this study was similar to a study involving 351 people in three counties in Great Britain (10.3%–[17]), as was the age structure of the knowledge (C. Seidel and K. Reinhardt, unpubl. data). Both studies were carried out with almost identical methodology. It is, therefore, possible, and testable, in future studies that 10–15% represents a current average for European industrialised countries. How good is that estimate? As all self-reports our study is limited as it rests on the respondents honesty and memory. However, we wish to point out that with respect to the identity of bed bug, dishonesty was impossible because we requested to know the identity. Also, some respondents had their last infestation a long time ago and the time since last contact with bed bugs was similar between those that did and did not recognize bed bugs.

One factor that may contribute to poor recognition rate is the out of context setting of the bed bug, being shown in a tube. It is possible that more people would have correctly guessed a bed bug if it had been placed in the bedroom, on a bed or on the wallpaper. Future studies can test how much the recognition, or guessing, ability of people would increase under more realistic settings. Future studies may also include other signs of an infestation such as eggs, nymphs or faecal stains, all of which are suspected to being less well recognised than the adult insect that we used. Finally, some studies in human behavioural ecology suggest an intrinsic ability of people to recognise parasites or other dangerous animals that evolved under fear [22], [24]. The responses assuming the bed bug to be a tick or a louse agree with that view, those assuming beetles or woodlice do not. If such a danger recognition exists and if the bed bug has been regarded as dangerous by our ancestors (as a non-vector the bed bug is not strictly dangerous), our data may contain false positives (people merely recognizing a parasite and falsely claiming it is a bedbug) as well as false negatives (people suggest these animals are fleas or ticks and in fact mean bedbugs). Unless more data exist, we suggest that the influence of an evolved fear may be small, or contain a similar number of false positives and false negatives.

The surveys were carried out in open public spaces, i.e. not settings that inspire confidentiality. Occasionally people replied to the question, “whether they have ever had contact with bed bugs in their lives”, in a reserved, indignant or sometimes even harsh way and insisted upon their cleanliness. Unfortunately, these responses were not quantified but it concerned an estimated 10% of the people, mostly elderly. About fifteen people were so upset they left the interviewer without finishing the questionnaire. If such firmly rooted stigma associated with bed bugs had resulted in people pretending not to know bed bugs, our overall estimate could be up to 10% too low.

The setting of the interview, public spaces in the inner city, does not allow us to speculate whether we had targeted a group that was, or was not, particularly knowledgeable about bed bugs. Future studies may focus on areas of known homogenous socioeconomic situations.

### Knowing about Bed Bugs

Most people reported they had heard about bed bugs but it was not possible to reveal the correctness of their answer. It is possible that some respondents actually referred to other creatures than bed bug. There is no German equivalent of the universally-known English saying: “Night, night, sleep tight, don’t let the bed bugs bite”. The colloquial ‘bug’ is not possible either to have contributed to any wrong reference (the German *Bettwanze* is a rather specific name) but it is likely, that people’s responses included references to insects or “vermin” other than bed bugs. For example, one respondent stated he knew bed bugs from Wilhelm Busch’s drawings (a famous German author and illustrator). However, Wilhelm Busch did not mention bed bugs in any of his stories (K. Reinhardt, unpubl. data), but he frequently mentioned fleas. Frequently people mentioned their surprise about the large size of bed bugs, as people assumed them to live in mattresses but this response was not quantified. These opinions suggest that some people think of dust mites. Future studies may include questions about bed bugs with which it becomes clearer whether people indeed think of bed bugs; such as asking whether they jump (then probably referring to fleas) or whether they live inside mattresses or pillows (then probably referring to dust mites).

In this study many people were interested in the bed bugs’ biology after they got to know their identity. Some people even thanked us for the opportunity to have a closer look at the insects. Unfortunately, the exact proportion was not quantified but estimated to be at least 10%. This concerned mainly younger people suggesting that any issue associating shame of bed bugs with a pretended lack of knowledge (see above) may be different for the age groups. We suggest that future questionnaires take these considerations into account.

Few studies exist that examine the efficacy of intervention means, mostly in a school [22] or an agricultural context [25], [26]. In our study, the internet was a minor source of knowledge about bed bugs. It was also an ineffective method. It is possible that this is related to lower marketing usage than in countries where the internet appears to be more important, such as the United States. Although reports seem to increase in frequency, websites about bed bugs in Germany are clearly not as widespread, prominent and intense as, for example, in the United States.

We found, perhaps unsurprisingly, that a 7-fold higher number of people identified bed bugs correctly if they had prior contact to bed bugs than those who had not. This suggests that showing bed bugs to people could dramatically improve their recognition for later occasions and so be an effective educational means. However, it is also important to note that a cohort of people who had seen or had contact with bed bugs, failed to recognize them later. Despite this, some of our results seem to indicate that presenting bed bugs as educational means may have a long-lasting effect over decades. It is currently impossible to estimate just how much educational means would improve recognition rates because having experienced bed bugs in one’s own home or the bed slept in is likely a more penetrating experience than being shown one. However, correcting for age, education was still almost twice as effective in recognizing a bed bug than no education (Table 1). Given the relatively low cost of implementing such education, it may be an effective way to increase people recognition ability of bed bugs. Given the interest particularly by young people into the biology of bed bugs, an effective way to improve knowledge and reduce irrational feelings about bed bugs might be to include some aspects into school curricula [22].

However, the follow-up question is perhaps more important: do people who correctly identify a bed bug react in a proper way to eliminate them?

### Getting Rid of Bed Bugs

That respondents were asked what they would do in case of a bed bug infestation is a hypothetical question and the correctness of the intention has not been tested here. Only 15% of the people would call a pest controller if they had a bed bug infestation but only 13% correctly identified the insect; another 5% mistook them for lice or cockroaches (i.e. 18%). If lice and cockroach presence elicit a similar proportion of people to call for pest controllers, an estimated 15% of the 18% (2.7%) call for correct professional help. In other words, and if these calculations are correct, a stunning 97% of the infestations in an initial stage may go undetected or untreated. However, this number probably, and hopefully, represents the lower limit.

If assuming that all respondents that self-educate themselves on the internet will contact a pest controller, this figure rises to a mere 3.1%. Only three additional scenarios make this estimate slightly more optimistic: If i) all respondents that take the bed bug for a tick consult their general practitioner (because of fear of tick-borne Lyme disease), and if ii) all practitioners correctly identify bed bugs and if iii) all practitioners recommend to call the pest controller, the number of people asking for correct help rises to 28%. This leaves 72% initial-stage infestations undetected. This figure may be too high, but note that its individual assumptions are testable. For example, the knowledge of bed bugs among GPs is unknown, but unlikely to be 100%. Similarly, it is unlikely that all 25% who identified the bed bugs as a tick would consult their physician.

In conclusion, we found evidence for a very strong misconception of bed bugs, both at the recognition level and at the potential action level. Some of the suggested measures could even make the situation problem more challenging to resolve.

## References

[1] Panagiotakopulu E, Buckland PC (1999) *Cimex lectularius* L., the common bedbug from Pharaonic Egypt. Antiquity 73: 908–911.

[2] Osborne PJ (1971) An insect fauna from the Roman site at Alcester, Warwickshire. Britannia 2: 156–165.

[3] Malotki E (1997) The bedbugs’ night dance and other Hopi tales of sexual encounter. London: Univ Nebraska Press.

[4] Soukand R, Kalle R, Svanberg I (2010) Uninvited guests: Traditional insect repellents in Estonia used against the clothes moth *Tineola bisselliella*, human flea *Pulex irritans* and bedbug *Cimex lectularius* . J Insect Sci 10 150: 1–18. 21070174 10.1673/031.010.14110 PMC3016901

[5] Potter MF (2011) The history of bed bug management – with lessons from the past. Am Entomol 57: 14–25.

[6] Doggett SL, Dwyer DE, Penas PF, Russell RC (2012) Bed bugs: clinical relevance and control options. Clin Microbiol Rev 25: 164–192. 22232375 10.1128/CMR.05015-11 PMC3255965

[7] Reinhardt K, Siva-Jothy MT (2007) Biology of bed bugs (Cimicidae). Annu Rev Entomol 52: 351–374. 16968204 10.1146/annurev.ento.52.040306.133913

[8] Doggett S, Russell RC (2008) The resurgence of bed bugs, *Cimex* spp. (Hemiptera: Cimicidae) in Australia. In Proceedings of the Sixth International Conference on Urban Pests, 13–16 July 2008, Budapest, Hungary. Robinson WH, Bajomi D (eds) Weszprém, Hungary, OOK-Press, 407–425.

[9] Reinhardt K (2012) Bedbug infestations. In: 2012 McGraw-Hill Yearbook of Science and Technology. New York: McGraw-Hill.

[10] Doggett SL, Geary MJ, Russell RC (2004) The resurgence of bed bugs in Australia, with notes on their ecology and control. Environm Health 4: 30–38.

[11] Richards L, Boase CJ, Gezan S, Cameron MM (2009) Are bed bug infestations on the increase within greater London? J Environm Health Res 9: 17–24.

[12] Harlan HJ, Faulde MK, Baumann GJ (2008) Bedbugs, 131–154 In Bonnefoy, X., Kampen, H. and Sweeney, K. 2008. Public Health Significance of Urban Pests. Copenhagen, Denmark: World Health Organization, Regional Office for Europe.

[13] Goddard J, deShazo R (2009) Bed bugs (*Cimex lectularius*) and clinical consequences of their bites. J Am Medic Assoc (JAMA) 301: 1358–1366. 10.1001/jama.2009.405 19336711

[14] Reinhardt K, Kempke D, Naylor R, Siva-Jothy MT (2009) Sensitivity to bedbug bites, *Cimex lectularius* . Med Vet Entomol 23: 163–166. 19292820 10.1111/j.1365-2915.2008.00793.x

[15] Leverkus M, Jochim RC, Schäd S, Bröcker E-B, Andersen JF, et al (2006) Bullous allergic hypersensitivity to bed bug bites mediated by IgE against salivary nitrophorin. J Invest Dermatol 15: 91–96. 10.1038/sj.jid.5700012 16417223

[16] Goddard J, deShazo R (2012) Psychological effects of bed bug attacks (*Cimex lectularius* L.). Am J Med 125: 101–103. 22195533 10.1016/j.amjmed.2011.08.010

[17] Reinhardt K, Harder A, Holland S, Hooper J, Leake-Lyall C (2008) Who knows the bedbug? Knowledge of the bedbug appearance increases with age in three counties of the UK. J Med Entomol 45: 956–958. 18826041 10.1603/0022-2585(2008)45[956:wktbbk]2.0.co;2

[18] Wang C, Saltzmann K, Chin E, Bennett GW, Gibb T (2010) Characteristics of *Cimex lectularius* (Hemiptera: Cimicidae), infestation and dispersal in a high-rise apartment building. J Econ Entomol 103: 172–177. 20214383 10.1603/ec09230

[19] King F (1990) ‘Mind the bugs don’t bite’. New Scientist 1701: 35–38.

[20] Reinhardt K, Isaac D, Naylor R (2010) Estimating the feeding rate of the bedbug *Cimex lectularius* in an infested room: an inexpensive method and a case study. Med Vet Entomol 24: 46–54. [Erratum: Med Vet Entomol 25: 116]. 10.1111/j.1365-2915.2009.00847.x 20377731

[21] Majekodunmi A, Howard MT, Shah V (2002) The perceived importance of cockroach [*Blatta orientalis* (L.) and *Blattella germanica* (L.)] infestation to social housing residents. J Environ Health Res 1(2): no pagination.

[22] Randler C, Hummel E, Prokop P (2012) Practical work at school reduces disgust and fear of unpopular animals. Society & Animals 20: 61–74.

[23] Seidel C (2011) A population analysis of *Cimex lectularius* (bed bug) in Germany during the 20th and 21st century: evidence of an increase of bed bug infestations in Germany. Zulassungsarbeit zur Wissenschaftlichen Prüfung für das Lehramt an Gymnasien in Baden-Württemberg. University of Tübingen.

[24] Prokop P, Fancovicova J, Fedor P (2010) Health is associated with antiparasite behaviour and fear of disease-relevant animals in humans. Ecolog Psychol 22: 222–237.

[25] Palis FG (1998) Changing farmers’ perceptions and practices: the case of insect pest control in central Luzon, Philippines. Crop Protection 17: 599–607.

[26] Nagaraju N, Venkatesh HM, Warburton H, Muniyappa V, Chancellor TCB, et al (2002) Farmers’ perceptions and practices for managing tomato leaf curl virus disease in southern India. Int J Pest Manag 48: 333–338.

